# Reservoir population ecology, viral evolution and the risk of emerging infectious disease

**DOI:** 10.1098/rspb.2022.1080

**Published:** 2022-09-14

**Authors:** Scott L. Nuismer, Andrew J. Basinski, Courtney Schreiner, Alexander Whitlock, Christopher H. Remien

**Affiliations:** ^1^ Department of Biological Sciences, University of Idaho, Moscow, ID 83844, USA; ^2^ Institute for Interdisciplinary Data Science, University of Idaho, Moscow, ID 83844, USA; ^3^ Bioinformatics and Computational Biology, University of Idaho, Moscow, ID 83844, USA; ^4^ Department of Mathematics and Statistical Science, University of Idaho, Moscow, ID 83844, USA

**Keywords:** infectious disease, spillover, evolution, reservoir, life history

## Abstract

The ecology and life history of wild animals influences their potential to harbour infectious disease. This observation has motivated studies identifying empirical relationships between traits of wild animals and historical patterns of spillover and emergence into humans. Although these studies have identified compelling broad-scale patterns, they are generally agnostic with respect to underlying mechanisms. Here, we develop mathematical models that couple reservoir population ecology with viral epidemiology and evolution to clarify existing verbal arguments and pinpoint the conditions that favour spillover and emergence. Our results support the idea that average lifespan influences the likelihood of an animal serving as a reservoir for human infectious disease. At the same time, however, our results show that the magnitude of this effect is sensitive to the rate of viral mutation. Our results also demonstrate that viral pathogens causing persistent infections or a transient immune response within the reservoir are more likely to fuel emergence. Genetically explicit stochastic simulations enrich these mathematical results by identifying relationships between the genetic basis of transmission and the risk of spillover and emergence. Together, our results clarify the scope of applicability for existing hypotheses and refine our understanding of emergence risk.

## 1. Introduction

We now live in a world where the consequences of viral emergence are woven into the day to day fabric of our lives. Remarkably, even while confronting this new reality on a daily basis, we have made little measurable progress developing the understanding we need to stop the next pandemic before it begins. A fundamental challenge we must confront is improving our ability to anticipate which viruses are likely to emerge and from which reservoir species (e.g. [1–4]). Recent progress in this direction has used statistical and machine learning methods to associate reservoir life-history traits or phylogenetic position with the presence of known viral pathogens (e.g. [2,5]). Similar methods have been used to learn which geographical regions are most likely to produce novel emerging viral infectious diseases (e.g. [6–8]). Hard-won progress has also been made by studying the detailed ecology of reservoirs and their viruses and using this information to generate more fine-scale predictions for when and where a particular virus is likely to spillover or emerge (e.g. [9–13]). Where we have made considerably less progress, however, is in understanding how evolution and genetic variation within viral populations shape the probability that spillover and/or emergence will occur.

One way viral evolution can shape the likelihood of spillover and emergence is by enabling adaptation to a novel host. The roots of this idea can be traced back (at least) to work in ecology studying how species can expand their geographical range and colonize a new niche [14,15]. Here, a central challenge is for evolution to increase the growth rate of the population within the new environment to self-sustaining levels before extinction occurs. At least in some circumstances, this process can be facilitated by repeated colonization events that introduce genetic variation capable of fuelling adaptation [14–16]. More generally, these ideas are captured by the idea of ‘evolutionary rescue’, where extinction of a poorly adapted population can be prevented by sufficiently rapid evolution [14,17]. Within infectious disease biology, these ideas were popularized and formalized in the paper by Antia *et al.* [18] which studied how spillover of an initially poorly adapted pathogen (*R*_0_ < 1) could lead to emergence if evolution was sufficiently rapid to reach self-sustaining levels of transmission (*R*_0_ > 1) before the pathogen population ‘stuttered’ to extinction. Although these ideas have been immensely helpful in understanding the process of emergence once spillover has occurred, they provide little guidance with respect to the viral pathogens most likely to evolve or the changes in reservoir ecology that might promote emergence.

Although much less well-explored, evolution may also act on viral pathogens within the reservoir in a way that predisposes them to successfully spillover and emerge [3]. This process of exaptation [19] may be linked to epidemiological dynamics within the reservoir population itself and may thus be sensitive to reservoir population ecology and life history. For instance, reservoir species with low population densities may support smaller and less genetically diverse viral populations that harbour fewer mutations capable of infecting humans. Alternatively, reservoir species with short lifespans and rapid population turnover may support larger and more genetically diverse viral populations that—by chance alone—include variants capable of infecting humans. Many other possibilities exist, and understanding the conditions that favour each requires that we understand how reservoir ecology and life-history influence viral epidemiology and evolution and, ultimately, the likelihood that human exapted viral genotypes are sampled by the human population through spillover [16].

Here, we develop a simple mathematical model that allows us to study how changes to reservoir population ecology and life-history influence viral epidemiology and evolution and the likelihood of viral spillover and emergence. Our model is predicated upon the assumption that adaptation to the human population requires genetic changes in the virus that are deleterious within the reservoir and is thus similar in spirit to the model analysed by Geoghegan *et al.* [3]. This assumption would hold if, for instance, the conformation of a coronavirus spike protein optimal for infection of the reservoir differs from the conformation optimal for infection of humans. We use this model to develop analytical predictions describing how reservoir lifespan alters the balance between mutation and selection within the reservoir and thus the frequency of mutations exapted to the human population and capable of sustained transmission and emergence. These analytical models are complemented by genetically explicit stochastic simulations that allow us to understand how the genetic basis of exaptation to the human host influences the likelihood of spillover and emergence.

## 2. The model

We model the epidemiological and evolutionary dynamics of viral spillover and emergence using a genetically explicit, multi-species extension of the classical susceptible (S), infected (I), recovered and immune (R) framework [20]. Our approach is similar to that used by others to study joint epidemiological and evolutionary dynamics [21–23]. We assume the virus circulates within a reservoir population living in sympatry with a human population and that these populations are well mixed. This scenario is inspired by infectious diseases such as Lassa virus that circulate within populations of rodents that regularly inhabit human dwellings where viral spillover into the human population is thought to occur [9,24,25]. Transmission is assumed to be density dependent within the reservoir and within the human population but allowed to be either density or frequency dependent from reservoir to human. Many forms of transmission other than these are, of course, possible [26].

### (a) Viral epidemiology and evolution within the reservoir

We assume the reservoir population has *per capita* rates of density-independent birth and death equal to *b* and *d*, respectively. Density dependence acts on both birth and death rates with strength defined by the parameters *α* and *ρ*, respectively. For simplicity, we assume the virus is avirulent within the reservoir and has no impact on mortality rate, although this need not always be the case [27]. The virus population is assumed to consist of *G* genotypes (indexed by *g*), each of which is characterized by a genotype-specific transmission rate within the reservoir/wildlife population (subscript *w*), *β*_*w*→*w*,*g*_, and a genotype-independent recovery rate, *γ*. Recovered individuals acquire immunity to the virus with this immunity waning at rate, *δ*. Mutation within the virus population is treated implicitly and assumed to occur within infected hosts and result in the wholesale conversion of a host infected with genotype *g*′ to a host infected with genotype *g* at a rate *μ*_*g*′→*g*_. With these assumptions, the population dynamics of the wildlife reservoir population and the epidemiological dynamics of the virus within the reservoir can be described by the following system of differential equations:

2.1a $$\frac{dS}{dt}=bN(1-\alpha N)+\delta R-S\sum_{g=1}^{G}\beta_{w\to w,g}I_{g}-(\rho N+d)S,$$

2.1b $$\frac{dI_{g}}{dt}=\beta_{w\to w,g}SI_{g}+\sum_{g^{\prime }=1}^{G}\mu_{g^{\prime }\to g}I_{g^{\prime }}-\sum_{g^{\prime }=1}^{G}\mu_{g\to g^{\prime }}I_{g}-(\gamma +d+\rho N)I_{g}$$

2.1c $$\mathrm{and}\;\frac{dR}{dt}=\gamma I^{*}-(\delta +d+\rho N)R,$$

where $N=S+\sum_{g=1}^{G}I_{g}+R$ defines the total density of reservoir individuals within the population, $I^{*}=\sum_{g=1}^{G}I_{g}$ defines the total density of infected reservoir individuals, and the subscript *w* → *w* indicates transmission is from one animal (‘wildlife’) to another.

### (b) The genetic architecture of spillover and emergence

**Figure 1.**
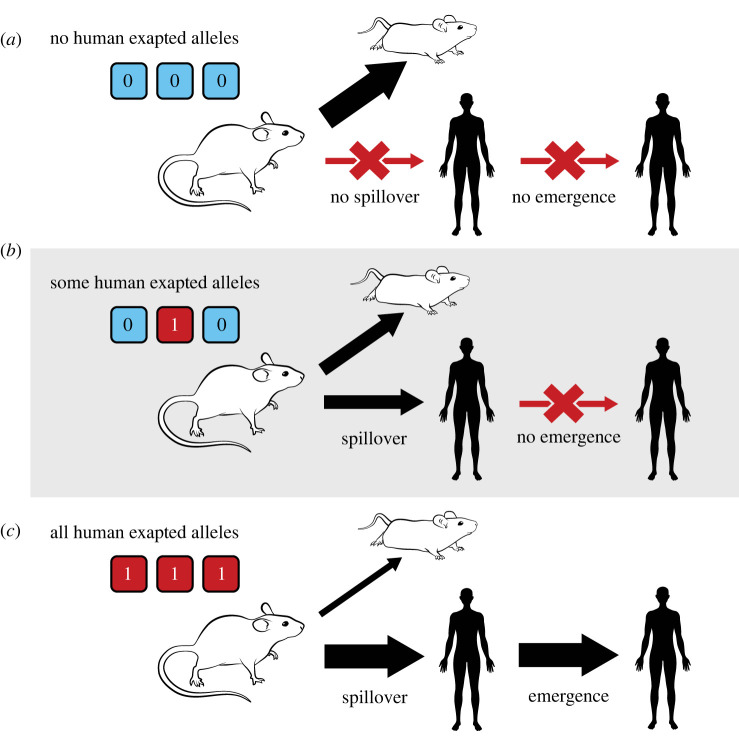
The genetic architecture of transmission, spillover and emergence for an example where three loci control transmission within and among species and three ‘1’ alleles are required to achieve levels of transmission within the human host sufficient for *R*_0_ > 1 and thus emergence. (Online version in colour.)

A central assumption of our approach is that some subset of virus genes have pleiotropic effects on infection of reservoir hosts and infection of human hosts. Specifically, we assume that alleles favourable for efficient transmission within the reservoir tend to be disfavourable for infection of humans and transmission among humans. This might occur, for instance, if the optimal conformation of a glycoprotein used for cell entry in the reservoir binds only poorly to human cells. We model this trade-off by assuming that the pathogen genome consists of *n* diallelic loci, where each locus carries either a ‘0’ or a ‘1’ allele. We assume that ‘0’ alleles improve the viruses’ ability to infect the reservoir but that ‘1’ alleles improve the viruses ability to infect humans. Thus, a viral genome consisting of primarily ‘0’ alleles will be good at infecting the reservoir but poor at infecting humans whereas a viral genome consisting of primarily ‘1’ alleles will be good at infecting humans but poor at infecting the reservoir (figure 1). Using the indicator variable *X*_*g*,*i*_ to define the allelic state of genotype *g* at locus *i* allows us to formalize these assumptions as expressions for the transmission rates within the reservoir, from reservoir to human and from human to human:

2.2a $$\beta_{w\to w,g}=\beta_{0}\prod_{i=1}^{n}(1-c_{w,i}X_{g,i}),$$

2.2b $$\beta_{w\to h,g}=\omega \beta_{0}\prod_{i=1}^{n}(1-c_{h,i}(1-X_{g,i}))$$

2.2c $$\mathrm{and}\;\beta_{h\to h,g}=\kappa \beta_{0}\prod_{i=1}^{n}(1-c_{h,i}(1-X_{g,i})),$$

respectively. The products in these expressions are taken over the *n* genetic loci involved in transmission (e.g. cell binding, tissue tropism, shedding, etc.), with the terms *c*_*w*,*i*_ and *c*_*h*,*i*_ quantifying the multiplicative loss in transmission to an animal or human host, respectively, that accrues to the virus for each maladaptive allele it carries. Tuning these parameters allows us to consider scenarios ranging from an absolute trade-off between performance in animal and human hosts to the complete absence of a trade-off. Adjusting these parameters also allows us to consider scenarios where trade-offs are driven by a single locus of major effect or where many loci contribute weakly. Finally, the parameters *ω* and *κ* scale the baseline transmission rate, *β*_0_, defined within the reservoir, to account for differences in encounter rates between reservoir and human populations and within the human population, respectively.

### (c) Quantifying the risk of spillover and emergence

We quantify the risk of spillover by defining the force of infection from the reservoir population into the human population:

2.3 $$\lambda_{\mathrm{Spill}}=\frac{I^{*}}{N^{k}}\sum_{g=1}^{G}\beta_{w\to h,g}x_{g},$$

where I* is the total number of reservoir individuals infected with the virus, *x*_*g*_ is the frequency of virus genotype *g* among infected reservoir individuals, *k* determines whether spillover is density (*k* = 0) or frequency (*k* = 1) dependent and the summation is taken over all virus genotypes. The extent to which the virus is able to infect humans is captured by the summation which explicitly calculates the expected transmission rate of the virus population into the human population.

We quantify the risk of emergence similarly to spillover, but restrict the summation to include only the subset, **E**, of virus genotypes for which *R*_0_ > 1 within the human population:

2.4 $$\lambda_{\mathrm{Emerge}}=\frac{I^{*}}{N^{k}}\sum_{g\in E}\beta_{w\to h,g}x_{g}.$$

Restricting the summation to those virus genotypes with *R*_0_ > 1 within the human population quantifies the force of infection for the subset of virus variants capable of generating self-sustaining epidemics, or emergence, within the human population. Results derived in the electronic supplementary material, appendix S1 demonstrate that the risk of emergence quantified by equation (2.4) is directly proportional to the probability that a sustained epidemic will develop within the human population in any period of time. Because this definition of emergence ignores the stochastic loss of many emergence-capable viruses, however, the rate at which spillover seeds an epidemic is only a fraction $1-(1/{\bar{R}}_{0})$ of this quantity where ${\bar{R}}_{0}$ is the average value of *R*_0_ within the human population for the subset of mutations with *R*_0_ > 1 (electronic supplementary material, appendix S1).

## 3. Results

### (a) Reservoir population ecology and life history drive the risk of viral spillover

We begin our analyses by studying how the population ecology and life history of the reservoir species defines the risk of spillover into the human population in the absence of evolution. Our goal is to develop the ecological backdrop against which the influence of evolution can be better understood. To this end, we study equations (2.1) in the limiting case of a genetically uniform virus capable of infecting humans and quantify the force of spillover into the human population when the reservoir population is at steady-state. We focus our attention on the rate of population turnover within the reservoir species, *T*, and how changing this rate—and thus the average lifespan of the reservoir—influences the risk of spillover. Formally, the rate of population turnover within our model can be found by solving for the *per capita* birth (or death) rate of the reservoir population when it is at its equilibrium population size, yielding the following expression:

3.1 $$T=\frac{b(d\alpha +\rho )}{b\alpha +\rho }.$$

As the rate of population turnover increases, average lifespan (1/*T*) decreases. Because we are interested in comparing species with different lifespans that are otherwise equally affected by the virus (in terms of recovery or waning immunity rates, i.e. epidemiologically interchangeable), we present key results in terms of a re-scaled rate of recovery from viral infection Γ=γ/T and a re-scaled rate at which immunity to viral infection wanes over time Δ = *δ*/*T*. These definitions, in conjunction with the equilibrium solutions to equations (2.1), allow us to quantify the force of infection from the reservoir population into the human population using equation (2.3):

3.2 $$\lambda_{\mathrm{Spill}}=\omega T\left(\frac{\left(R_{0}-1)(1+\Gamma )(1+\Delta \right)}{1+\Gamma +\Delta }\right){\left(\frac{1}{\hat{N}}\right)}^{k},$$

where *k* is equal to 0 if spillover into the human population is density dependent and 1 if frequency dependent, $\hat{N}$ is the steady-state population size of the reservoir, and *R*_0_ is the expected number of new viral infections caused by a virus infected reservoir individual when introduced into a completely susceptible reservoir population (electronic supplementary material, appendix S2). Here, *R*_0_ is equal to:

3.3 $$R_{0}=\frac{\beta (b-d)}{\rho (b+\gamma )+\alpha b(\gamma +d)}.$$

Unsurprisingly, the risk of spillover increases with *R*_0_.

**Figure 2.**
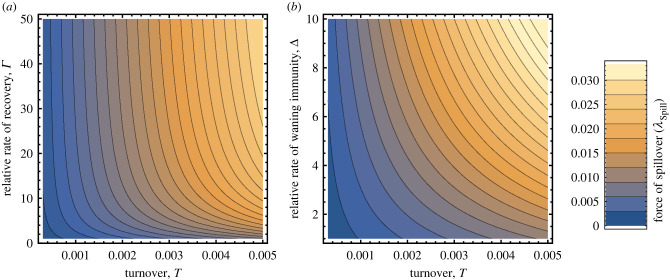
The force of spillover into the human population as a function of reservoir population turnover rate and the relative recovery rate from viral infection (*a*) and as a function of reservoir population turnover rate and the relative rate at which virus immunity wanes (*b*). In (*a*) Δ was set to 5 and in (*b*) Γ was set to 25. Viral *R*_0_ was held constant at a value of 2.0 in both panels, *ω* was set equal to 1, and spillover was density dependent such that *k* = 0. The figure was generated by plotting equation (3.2) using *Mathematica*. (Online version in colour.)

As demographic turnover increases and average reservoir lifespan (1/*T*) decreases, result (3.2) shows that the force of spillover increases (figure 2). There are multiple explanations for this effect, the most important of which depends on what else is held constant. For instance, if we wish to compare across species with different rates of turnover but identical population sizes, the effect can be attributed to increased levels of pathogen transmission within the reservoir population. This necessitates, however, that either the density-independent death rate, *d*, or density dependent death rate *ρ* rises with turnover. If, by contrast, we wish to compare species with identical values of *d* and *ρ*, the effect can be attributed to the association between rapid rates of population turnover and increases in total reservoir population size. No matter the precise underlying mechanism at play, however, the result is consistent with the ‘pace of life’ hypothesis and supports the idea that short-lived species with rapid population turnover may be particularly potent sources of spillover into the human population [5,28–30].

The terms Γ=γ(1/T) and Δ = *δ*(1/*T*) that appear in equations (3.2) and (3.3) measure the recovery rate from virus infection (Γ) and the rate at which immunity wanes (Δ) relative to the expected lifespan of the reservoir, 1/*T*. Thus, a value of Γ=2, for instance, indicates that the average recovery time from virus infection is half the average reservoir lifespan. As long as *R*_0_ is held constant, the force of spillover increases as a function of both recovery rate and the rate at which immunity is lost, suggesting that infections generating short-term acute infections with short-term immunity provide the greatest opportunities for spillover (figure 2).

### (b) Viral evolution modulates the risk of emergence

Before diving into more complex evolutionary and genetic scenarios using simulations, we illustrate several key factors influencing the likelihood of viral emergence using a single locus model and analytical approximations. Specifically, we assume the rate of viral transmission from reservoir to reservoir, from reservoir to human and from human to human depends on only a single diallelic genetic locus (e.g. a change in a single nucleotide that alters the conformation of a coronavirus spike protein) with possible alleles H or h. We further assume that only one of these two alleles (the H allele) is capable of generating sustained human-to-human transmission (*R*_0_ > 1) and thus emergence. This assumption greatly simplifies the model and allows evolutionary change within the reservoir to be described by

3.4 $$\frac{dp}{dt}=-c_{w}\beta_{0}p(1-p)S+\mu (1-2p),$$

where *μ* is the rate of (symmetric) mutation between H and h alleles, *c*_*w*_ is the reduction in transmission experienced by viral pathogens carrying the human exapted H allele within the reservoir, and *p* is the frequency of the H allele that is maladaptive for transmission within the reservoir population but exaptive for transmission to and among humans (electronic supplementary material, appendix S3). Although simplistic, equation (3.4) demonstrates that the strength of selection acting against the human exapted allele within the reservoir population is *c*_*w*_*β*_0_*S* such that selection increases as the density of susceptible individuals within the reservoir population grows. This occurs because a large pool of susceptible individuals increases opportunities for transmission within the reservoir and thus the relative increase in growth rate experienced by the reservoir adapted allele. From the perspective of viral emergence, this simple result has an important consequence: the weaker selection against the human adaptive allele within the reservoir population, the greater its frequency when a balance between selection and mutation is reached. Thus, we expect that reservoir populations with a low density of susceptible individuals will harbour a greater frequency of human exapted mutations than reservoir populations with a high density of susceptible individuals.

Solving for the equilibrium frequency of the human exapted allele when it is rare within the reservoir population (electronic supplementary material, appendix S3), yields the following approximate expression for its frequency:

3.5 $$\hat{\;p}\approx \frac{\mu }{c_{w}((1+\Gamma )T+\mu )}.$$

**Figure 3.**
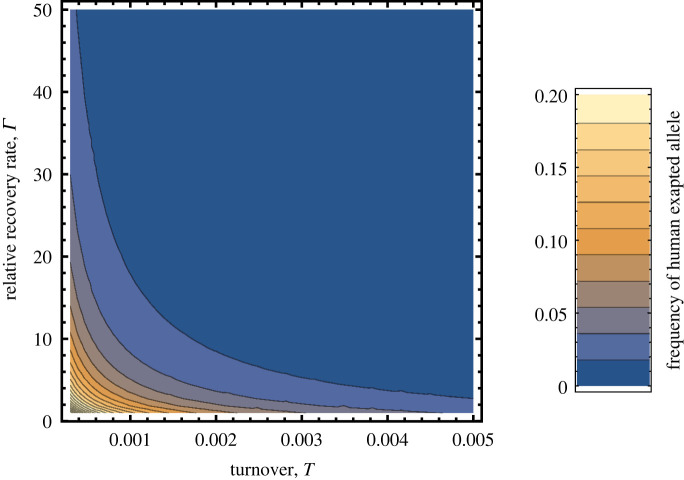
The frequency of the exapted allele that confers high transmission to and within the human population as a function of reservoir population turnover rate and the recovery rate from virus infection. The viral mutation rate, *μ* was set equal to 0.0001 and the cost of carrying the human exapted allele within the reservoir population, *c*_*w*_, was set equal to 0.9. virus *R*_0_ was set equal to 2.0. The plot was generated using equation (3.5) and *Mathematica*. (Online version in colour.)

Result (3.5) illuminates how reservoir ecology and life history interact to shape the frequency of the human exapted mutation and thus opportunities for viral emergence. Specifically, we see that the frequency of the allele conferring the ability to transmit well within the human population falls as the rate of demographic turnover within the reservoir, *T*, rises. This occurs because as population turnover increases, so too does the density of susceptible individuals within the reservoir population. This, in turn, strengthens selection for the allele which increases transmission within the reservoir. By contrast, low population turnover reduces the density of susceptible individuals and opportunities for transmission within the reservoir, thus weakening selection against the allele exapted for human transmission. As a consequence, the balance between mutation and selection within the reservoir shifts, and the frequency of the allele conferring efficient transmission within the human population rises (figure 3). In addition to the rate of turnover within the reservoir population, result (3.5) demonstrates that the frequency of the allele conferring efficient transmission within the human population increases as the duration of the viral infectious period increases (figure 3). This occurs primarily because prolonged infections increase opportunities for mutation from the allele conferring high transmission within the reservoir population to the exapted allele that confers high transmission to and within the human population. Finally, result (3.5) confirms the easily anticipated results that the frequency of the allele conferring high performance within the human population rises with the viral mutation rate *μ* and falls with the selective cost this allele imposes on transmission within the reservoir population *c*_*w*_. Comparing these results with those from the previous section reveals that the rate of turnover within the reservoir population and the recovery rate from virus infection have qualitatively different impacts on ecological and evolutionary risk (compare figures 2 and 3). In the next section, we integrate ecology and evolution to resolve the net impact of turnover and recovery rates on the force of emergence for the simple diallelic model we have considered here.

### (c) Emergence risk depends on the balance between spillover and evolution

To derive the most transparent results possible, we again focus on the simple scenario described in the previous section where a single diallelic locus controls transmission within and among species and only the human exapted allele H is capable of sustained human to human transmission (*R*_0_ > 1 within the human population) and thus emergence. For simplicity, we assume the human exapted allele H is maintained at a low frequency within the reservoir population at a balance between selection and mutation. This assumption should hold anytime the allele conferring improved transmission to and among humans is strongly deleterious with respect to transmission within the reservoir population and mutation rates are not extreme. With this assumption, the steady-state solution for the force of emergence is

3.6 $$\lambda_{\mathrm{Emerge}}\approx \omega \frac{1+\Delta }{1+\Delta +\Gamma }\left(\frac{\mu }{c_{w}}(R_{0}-1)\right){\left(\frac{1}{\hat{N}}\right)}^{k},$$

where *k* is equal to 0 if spillover into the human population is density dependent and 1 if frequency dependent and $\hat{N}$ is the steady-state population size of the reservoir (electronic supplementary material, appendix S4). Equation (3.6) is an accurate approximation as long as the strength of selection, $c_{w}T(1+\Gamma )$, is much larger than the rate of mutation: $c_{w}T(1+\Gamma )>>\mu$. The more cumbersome exact result is provided within the electronic supplementary material, appendix S4.

**Figure 4.**
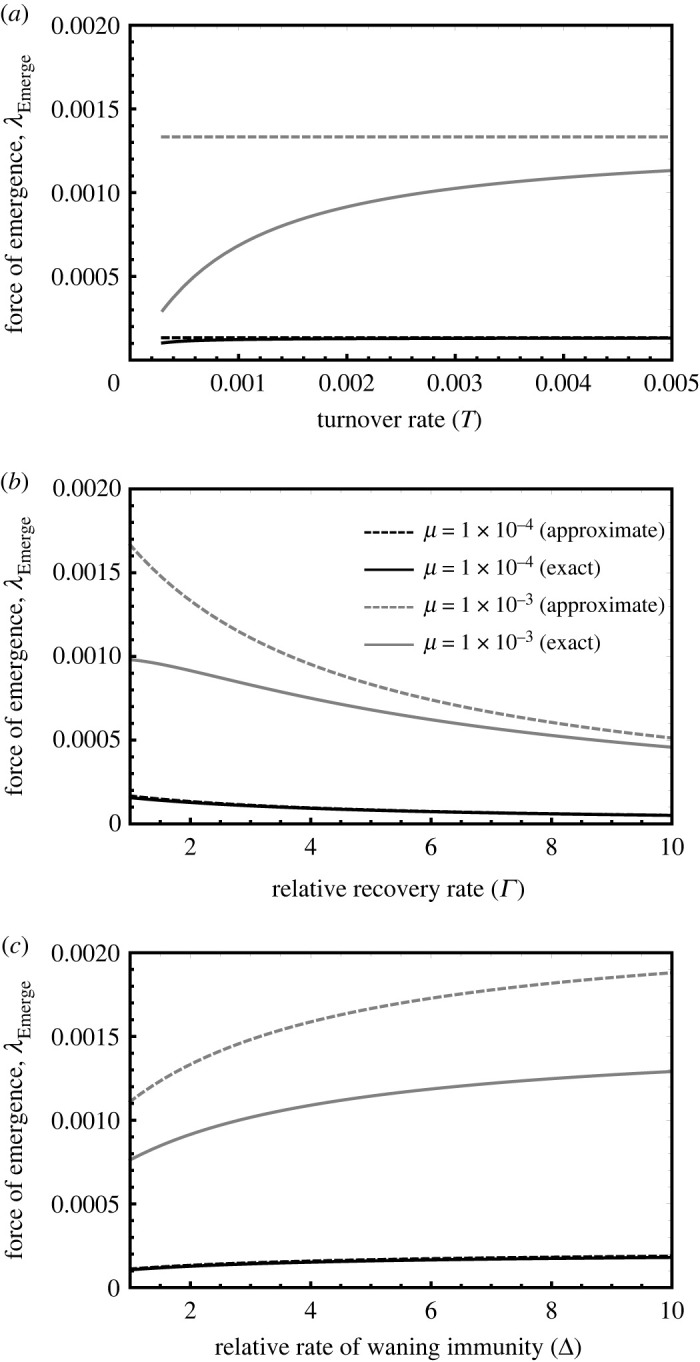
The force of emergence predicted by approximation (3.6) (dashed lines) and the exact solution given by equation (38) of the electronic supplementary material, appendix S4 (solid lines), as a function of reservoir population turnover (*a*), virus recovery rate within the reservoir (*b*) and rate at which immunity wanes within the reservoir population (*c*). Background parameters used in common across all panels were *R*_0_ = 3, *c*_*w*_ = 0.9 and *ω* = 1. Parameters specific to each panel were Γ=2, and Δ = 2 in the top panel, *T* = 0.002 and Δ = 2 in the middle panel and *T* = 0.002 and Γ=2 in the bottom panel. All plots were performed using *Mathematica*. Note that the poor fit of the approximation in panel (*a*) was expected for the high rate of mutation shown.

Five important findings emerge from approximation (3.6). First, the rate of population turnover within the reservoir does not influence emergence risk as long as the approximation holds (figure 4*a*). This occurs because the impact of population turnover on the ecology of spillover precisely cancels with the impact of turnover on viral evolution. Thus, although rapid population turnover increases the force of spillover, it also increases selection against the human exapted mutation by increasing the importance of transmission within the reservoir population. As the mutation rate rises and the human exapted allele begins to reach appreciable frequencies, this approximation begins to break down and the ecological impacts of population turnover begin to outweigh the evolutionary impacts (figure 4*a*). As a consequence, high rates of mutation set the stage for increasing rates of population turnover to increase emergence risk through its impact on viral prevalence.

Second, viral pathogens that cause acute short-term infections (large values of Γ) within the reservoir are less likely to lead to emergence (figure 4*b*). This occurs because rapid recovery decreases the prevalence of infection within the reservoir population and reduces opportunities for mutation to occur within the reservoir host. Third, viruses for which immunity within the reservoir population is short-lived (large values of Δ) are more likely to seed emergence (figure 4*c*). This occurs because increasing the rate at which immunity wanes increases the prevalence of the virus within the reservoir population. Fourth, the force of emergence increases with mutation rate (*μ*) and decreases with the strength of selection within the reservoir population (*c*_*w*_). Finally, the force of emergence always increases with virus *R*_0_ within the reservoir.

### (d) Emergence risk depends on the genetic basis of host adaptation

To evaluate the generality of our single locus results, we developed and analysed stochastic simulations of our model for scenarios where virus transmission rates depend on more than a single locus. These simulations allow us to compare cases where a single nucleotide substitution is sufficient for emergence to cases where multiple nucleotide substitutions are required. In addition, these simulations allow us to integrate stochastic effects that may be particularly relevant for the rare deleterious mutations we study here, and which we assume fuel emergence through chance exaptation to the human host. Our simulation approach makes use of the Gillespie algorithm with a tau leaping approximation to rapidly simulate model (2.1) for scenarios in which transmission within the reservoir, from reservoir to human and among humans depends on up to 10 diallelic genetic loci. We assume perfect pleiotropy such that all loci involved in determining virus transmission within the reservoir are also involved in determining virus transmission to humans and among humans. Mutation is modelled by assuming reservoir hosts infected with strain *g* convert to one of the *n* neighbouring strains with genotype *g*′ at rate *μ*. Our approach ignores the complexities of recombination within the viral genome.

Simulations were run for a burn-in period of five years and all spillover and emergence events were then tracked over the following five years for a broad range of parameter combinations. The burn-in and study periods of five years correspond to between 200 and 3333 lifespans for the range of population turnover rates we considered. Initial simulation testing demonstrated this was sufficient to reach a quasi-steady state. Spillover events occurred at rate *λ*_Spill_ × *H* where *H* is the density of the human population and *λ*_Spill_ was calculated using equation (2.2*b*) for cases where between 1 and 10 loci determined transmission rates. Emergence occurred in the subset of spillover events where the virus genotype that spilled over had an *R*_0_ > 1 within the human population calculated using equation (2.2*c*). Because this definition of emergence ignores the stochastic loss of many emergence-capable viruses, the rate at which spillover seeds an epidemic is only a fraction $1-(1/{\bar{R}}_{0})$ of this quantity where ${\bar{R}}_{0}$ is the average value of *R*_0_ within the human population for the subset of mutations with *R*_0_ > 1 (electronic supplementary material, appendix S1). Spillover and emergence events were then averaged over the five years post-burn-in and the average number of spillovers and emergence events per day used in all analyses.

**Figure 5.**
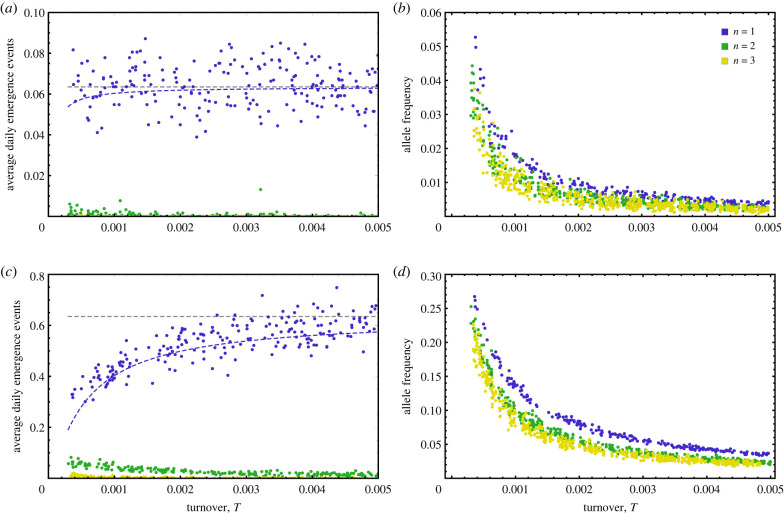
The average number of daily emergence events as a function of population turnover (left column) and the frequencies of human exapted alleles as a function of population turnover (right column) for the case where a single genetic locus mediates transmission (blue), two loci mediate transmission (green) and three loci mediate transmission (yellow). Each dot represents the average number of emergence events per day for a single stochastic simulation. The grey dashed line shows the analytical approximation for the predicted number of emergence events each day (equation (3.6)) and the blue dashed line the exact prediction for daily emergence events. In the first row, the mutation rate is weak (*μ* = 0.0001) and our approximation performs well. In the second row, the mutation rate is tenfold larger (*μ* = 0.001) than in the first row and, as expected, the approximation begins to break. Parameter values were *R*_0_ = 2.0, Γ=5.0, Δ = 1.0, *c*_*w*_ = 0.9, *c*_*h*_ = 0.5. The human population size was set to 2000 and the value of *κ* set such that the most transmissible virus genotype within the human population had an *R*_0_ = 1.2 within the human population. Transmission from reservoir to human was assumed to be density dependent such that *k* = 0. Figure panels were generated by plotting equation (3.6) and simulated data using *Mathematica*. (Online version in colour.)

We investigated how the genetic basis of transmission influenced the risk of spillover and emergence by assuming the difference in transmission rate between the worst and best genotypes was identical for scenarios involving different numbers of loci. Thus, we assume that the effect of each locus on transmission is reduced as the number of loci is increased. Under these conditions our simulations demonstrate that increasing the number of loci reduces the risk of emergence (figure 5).

This result arises because emergence requires sampling a virus genotype carrying human exapted alleles at an increasingly large number of loci. Because the human exapted alleles are deleterious within the reservoir—and thus rare—the greater the number of loci, the lower the probability of drawing the required combination of human exapted alleles. Although important, this result is rather intuitive. If the mutation rate becomes appreciable, however, a less easily anticipated relationship develops between the genetic basis of transmission and the influence of population turnover on emergence risk. Specifically, if mutation rates are appreciable but only a single locus determines rates of transmission, increasing the rate of population turnover increases the risk of emergence (figure 5*c*; blue dots). As the number of loci determining transmission increases, however, this relationship reverses and increasing rates of population turnover result in decreased emergence risk (figure 5*c*; orange and green dots). The reason for this shift is the increasing importance of the evolutionary component of emergence risk as the number of loci increases.

## 4. Discussion

We have developed and analysed simple mathematical and computational models that couple epidemiological and evolutionary dynamics within a reservoir population to the risk of spillover and emergence into the human population. Our models rest on the biologically plausible assumption that viral genotypes optimized for transmission within the reservoir are unlikely to be simultaneously optimized for transmission into and within the human population [31]. Thus, virus genotypes that are exapted to the human population will persist within the reservoir population at relatively low frequencies determined by the balance between selection and mutation. Because the strength of selection acting against human exapted genotypes depends on epidemiological dynamics within the reservoir as well as reservoir ecology and life history, our results allow us to formalize existing verbal arguments and clarify the conditions under which they apply. Perhaps most importantly, our results show that blanket arguments for reservoir characteristics associated with emergence risk greatly oversimplify the complex relationships that determine when and where spillover and emergence are most likely to occur.

Numerous studies have pursued the idea that the life-history traits of a species can be used to assess the likelihood that they serve as reservoir for viral pathogens capable of spillover and emergence [2,29,32]. One particularly popular and common argument is that reservoir species with a fast-paced lifestyle are more likely to seed spillover and emergence events [28,29]. Our mathematical solutions demonstrate that this argument holds rigorously anytime virus genotypes are epidemiologically interchangeable. Specifically, our results show that in such cases, the risk of spillover rises as the rate of population turnover increases. Because the rate of population turnover is the reciprocal of realized lifespan, these results support arguments that short-lived species are more likely to serve as reservoirs for emerging infectious disease. When virus genotypes are not epidemiologically interchangeable and instead differ with respect to rates of transmission, however, our results show that this appealingly simple argument crumbles. The reason this occurs is that, once virus genotypes differ with respect to their rates of transmission, evolutionary dynamics begin to contribute to the risk of spillover and emergence. In general, our results show that increasing rates of population turnover cause evolution to reduce the risk of spillover and emergence. Thus, population turnover increases the risk of spillover but decreases the frequency of virus genotypes that are capable of successfully infecting and transmitting among humans. Ultimately, then, the consequences of population turnover for spillover and emergence risk depend on the balance between ecological and evolutionary forces. How these forces balance out depends on the rate of mutation and the strength of selection acting against human exapted genotypes within the reservoir population and also the genetic basis of exaptation.

In addition to the rate of population turnover, our results suggest that epidemiological properties of viruses within the reservoir may be important determinants of spillover and emergence risk. For instance, our results demonstrate that viral pathogens yielding short-term acute infections are less likely to lead to emergence than viruses that generate long-term persistent infections. The reason this effect arises is that our model assumes selection within the reservoir acts on transmission alone. Thus, long-term persistent infections offer far greater opportunities for mutation to increase the frequency of the deleterious alleles that confer exaptation to the human host than do short-term acute infections that experience more frequent purging selection. Although sufficient data is not yet available to robustly test this prediction, it is consistent with observed persistent viral infection in bats and the rapid accumulation of SARS-CoV spike mutations during persistent *in vitro* infection [31,33–35]. Our results also demonstrate that the durability of virus immunity within the reservoir population influences spillover and emergence risk. Specifically, we find that risk increases with the rate at which immunity is lost. In this case, the driving force is epidemiological rather than evolutionary, with increasing rates of waning immunity leading to a greater viral prevalence within the reservoir population but no change in the frequency of human exapted mutations.

Our exploration of the genetic basis of spillover and emergence revealed two important results. First, as the number of loci involved in exaptation to the human host increases, the likelihood of emergence drops. This result is unsurprising as our model assumes emergence requires a virus strain carrying multiple rare alleles to spillover. As the number of loci involved in exaptation to the human host rises, the probability of sampling a virus strain carrying an increasingly large number of rare alleles falls. Although this result is intuitive, it suggests a better understanding of the genetic mechanisms involved in viral adaptation to human hosts would facilitate efforts to predict emergence risk [36]. The second result revealed by our multi-locus simulations is less intuitive and rooted in the interplay between epidemiological and evolutionary dynamics. Specifically, our results show that when the rate of population turnover has an impact on emergence risk, this impact tends to be positive when exaptation depends on only a single genetic locus but negative when larger numbers of loci are involved. The reason for this effect is the increasing importance of the evolutionary component of emergence risk—which causes human exapted alleles to decrease in frequency as the rate of turnover increases—as the number of loci rises. Thus, for those viral pathogens where sustained transmission among humans requires change in only a single nucleotide, the risk of emergence should be greater from reservoir species with a high rate of population turnover. By contrast, for viral pathogens where sustained transmission among humans requires multiple nucleotide changes, those reservoir species with a slow rate of population turnover should represent the greatest emergence risk. This nuanced relationship between the genetic mechanism of host adaptation and population turnover complicates efforts to predict the risk of emergence from reservoir species with different rate of population turnover or natural lifespans (e.g. [2]).

Although we have used a range of methods and analyses to ensure our predictions are robust, our results do rest on several important assumptions. Of these, we anticipate that four are the most likely to influence our predictions. First, we have assumed virus performance in reservoir and human populations is controlled by an identical set of genetic loci with directly opposing effects on fitness in each host. Although this is a strong assumption, it is consistent with some well-studied examples of viral adaptation such as the spike protein of SARS-CoV-2 where specific nucleotide substitutions are known to be associated with performance in different hosts (e.g. [31,37]). Even in cases where pleiotropy is not absolute, however, we anticipate that our results will continue to hold qualitatively. Second, our model assumes that genotypes differ only with respect to their rates of transmission within reservoir and human populations. If alleles that alter transmission also influence other epidemiologically relevant traits such as recovery rate, virulence, or duration of immunity, however, our results may break down. Although a substantial body of theory has focused on the consequences of such trade-offs for viral evolution [38], the ubiquity of alleles with pleiotropic effects on transmission and other epidemiologically traits remains largely unknown. Third, we have focused our analyses on steady-state solutions where vital rates of the reservoir population remain constant over time. If these vital rates change seasonally [11,39] or in response to anthropogenic manipulation or disturbance [40,41], our results may need to be adjusted or modified [42]. Finally, our models ignore the potential for multiple infection of individual hosts and recombination.

Understanding the relationships between the ecology and life history of wild animals and their potential to serve as reservoirs for viral pathogens capable of causing emerging infectious disease in humans is a fundamental challenge for our ability to anticipate—and potentially preempt—future pandemics. Existing studies have largely focused on identifying patterns and statistical relationships between reservoir traits and emergence risk without probing underlying mechanisms. We have taken an alternative approach to this challenge and used mechanistic models that couple reservoir population ecology, virus evolution and emergence risk to clarify when and why we expect these relationships to occur. By connecting mechanisms to predicted patterns, our work represents a first step in generating testable hypotheses that could better inform efforts to reduce the risk of emerging infectious disease.

## Data Availability

The data are provided in the electronic supplementary material [43].
